# Hypertrophic olivary degeneration secondary to brain abscess: a case report and literature review

**DOI:** 10.3389/fnhum.2025.1674100

**Published:** 2025-10-28

**Authors:** Shihan Tian, Biqiu Tang, Kai Zhang, Xiaohe Tian, Na Hu

**Affiliations:** ^1^Department of Radiology, West China Hospital of Sichuan University, Chengdu, China; ^2^Department of Radiology, Huaxi MR Research Center (HMRRC), Institution of Radiology and Medical Imaging, West China Hospital of Sichuan University, Chengdu, Sichuan, China; ^3^Psychoradiology Key Laboratory of Sichuan Province, West China Hospital of Sichuan University, Chengdu, Sichuan, China; ^4^Sichuan Provincial Engineering Research Center of Intelligent Medical Imaging, West China Hospital, Sichuan University, Chengdu, China

**Keywords:** hypertrophic olivary degeneration, brain abscess, magnetic resonance imaging, diffusion tensor imaging, case report

## Abstract

**Introduction:**

Hypertrophic olivary degeneration (HOD) is a rare transsynaptic neurodegenerative disorder arising from disruption of the Guillain-Mollaret triangle (GMT), a neural circuit critical for motor coordination. Classical clinical presentation includes palatal tremor. While cerebrovascular etiology dominates reported cases, HOD secondary to intracranial infections remains poorly characterized, posing diagnostic challenges.

**Case presentation:**

A 72-year-old man with diabetes and hypertension presented with a 7-day history of fever, headache, and vomiting. Initial neuroimaging revealed right cerebellar hematoma with multiple brain abscesses. Antibacterial treatment achieved symptomatic improvement, but follow-up was lost. Seven months later, readmission occurred due to memory decline and personality changes. Magnetic resonance imaging (MRI) showed T_2_-weighted hyperintensity in the left anterior medulla oblongata and hemosiderosis in the right cerebellar hemisphere. Despite the absence of clinical manifestations of HOD, prior abscess-induced GMT involvement strongly supported the diagnosis. Symptomatic management stabilized the patient, with persistent lesions but no clinical progression at 5-month follow-up.

**Conclusion:**

This case documents a rare case of HOD following bacterial brain abscess, presenting with atypical clinical features. It expands the etiological spectrum of HOD and underscores the need for heightened clinical suspicion in post-infectious neurological deterioration. Multimodal MRI facilitates early diagnosis and timely intervention, highlighting its critical role in managing this underrecognized entity.

## Introduction

Hypertrophic olivary degeneration (HOD) is a rare transsynaptic degenerative disease. Pathogenesis involves multi-etiological damage to the dentato-rubro-olivary pathway (Guillain-Mollaret triangle, GMT), a neural circuit critical for motor coordination. The hallmark clinical manifestation is palatal tremor (Marrakchi et al., 2024). Subtle initial symptoms often lead to missed diagnosis or misdiagnosis (Patay et al., 2014; Sechi et al., 2019). Identifying HOD provides a window for early intervention before debilitating symptoms arise (Ogut et al., 2023). While most frequently associated with cerebrovascular events (Gao et al., 2022), intracranial infection-induced HOD remains exceptionally uncommon. This report documents HOD development following brain abscess complicating meningitis, highlighting diagnostic complexities and circuit-level pathomechanisms.

## Clinical and imaging data

**Figure 1:**
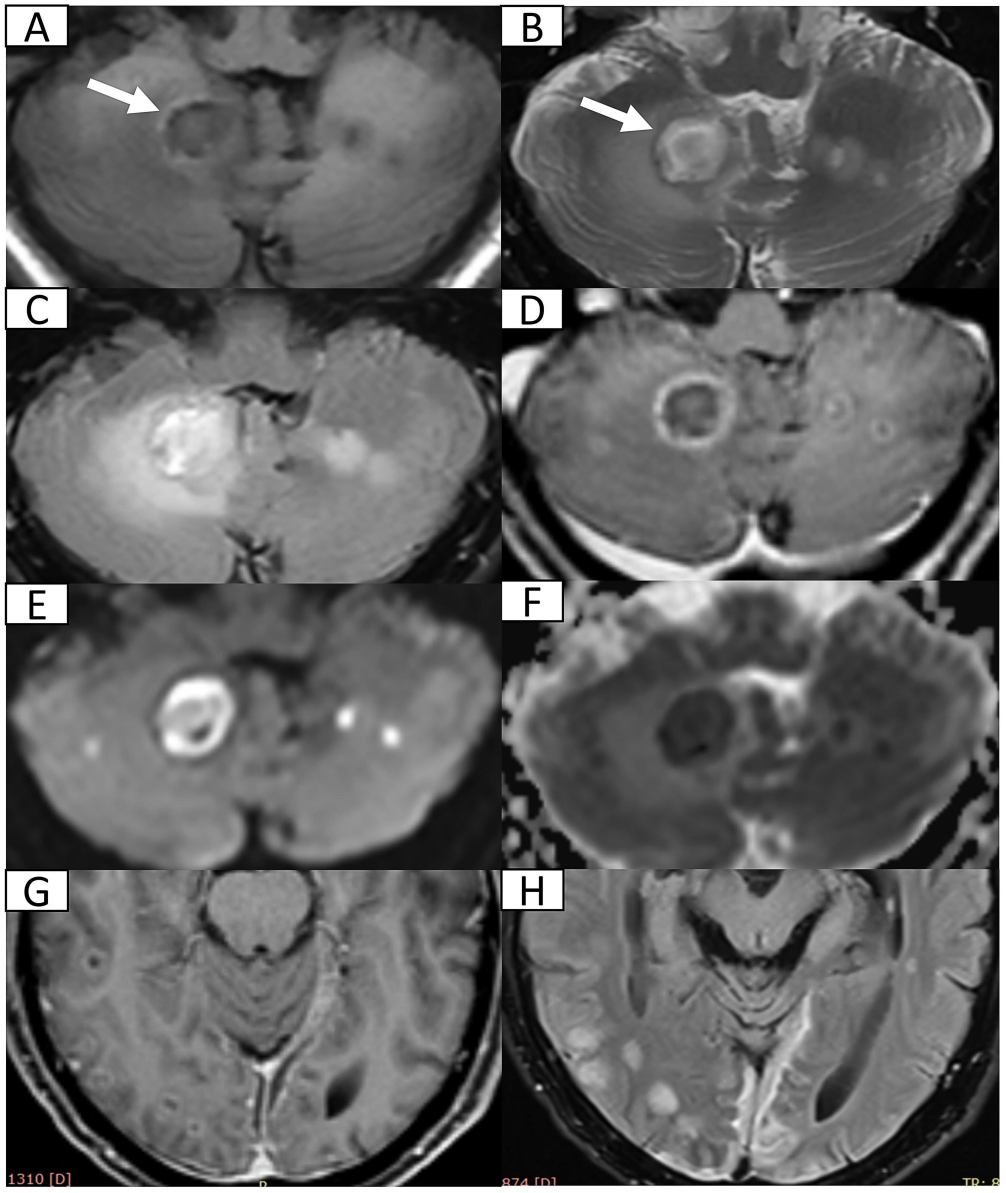
Head 1.5 T MRI after first admission. **(A,B)** Axial T_1_-weighted imaging (T_1_WI) and T_2_-weighted imaging (T_2_WI) reveal multiple cerebellar nodules and subacute hemorrhage (arrow) in the right cerebellum. **(C)** Axial fluid-attenuated inversion recovery (FLAIR) sequence demonstrates multiple hyperintense nodules with surrounding vasogenic edema. **(D)** Post-contrast axial T_1_WI confirms ring-enhancing abscesses in bilateral cerebellum. **(E,F)** Axial diffusion weighted imaging and corresponding apparent diffusion coefficient map indicate restricted diffusion within abscess cavities suggestive of viscous purulent content. **(G)** Post-contrast axial T_1_WI at the pontine level shows disseminated supratentorial infection including ring-enhancing abscesses in bilateral occipital and right temporal lobes and leptomeningeal enhancement (meningitis). **(H)** Axial FLAIR sequence demonstrates crescentic hyperintensity along cerebral convexities indicating subdural empyema.

A 72-year-old male with type 2 diabetes and hypertension presented with a 7-day history of fever, headache, and vomiting. Notably, blood glucose was not routinely monitored. Five years prior, he had discontinued smoking and alcohol consumption. Initial head computed tomography (CT) revealed a right cerebellar hematoma with multiple hypodense lesions showing partial rim enhancement (Supplementary Figures S1A–C). CT angiography excluded vascular abnormalities (Supplementary Figure S1D). Upon admission, he was alert with dysarthria, dysphagia, and unsteady gait, but preserved cognition. No prior history of unconsciousness, visual abnormalities, aspiration, seizures, or facial nerve dysfunction. Physical exams showed bilaterally responsive pupils (3 mm), normal muscle strength and tone, and no pathological reflexes. Vital signs revealed fever (38.8 °C) and blood tests showed mild leukocytosis (10.0 × 10^9^/L). Head magnetic resonance imaging (MRI, 1.5 T) with diffusion-weighted imaging (DWI) and contrast enhancement confirmed multiple brain abscesses, hemorrhagic cerebellar lesion, meningitis, and subdural empyema (Figure 1), establishing complicated intracranial infection diagnosis. Management included supportive care [oxygen, intravenous (IV) fluids, expectorants, electrolyte correction, and glycemic and hypertensive control], antiemetics (dolasetron mesylate 12.5 mg IV bid and ondansetron 8 mg IV bid), and antimicrobials (cefoperazone 4 g IV q12h and linezolid 600 mg IV q12h). Symptoms resolved within 7 days. He was discharged with instructions for 1-month follow-up MRI but was lost to follow-up.

**Figure 2:**
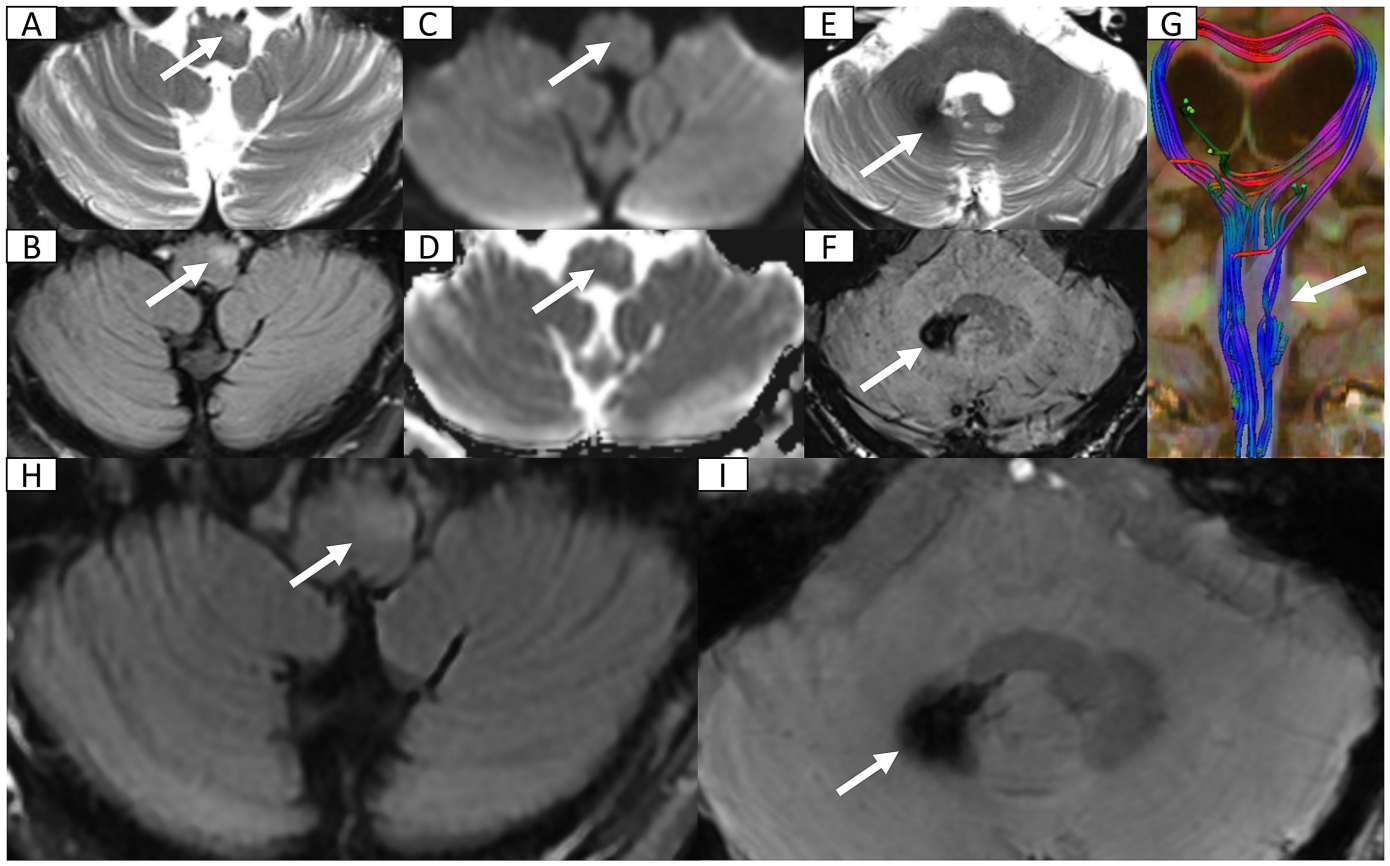
Head 3 T MRI after second admission and at 5-month follow-up. **(A,B)** Axial T_2_-weighted imaging (T_2_WI) and fluid-attenuated inversion recovery (FLAIR) sequence reveal focal hyperintensity and mild hypertrophy in the left anteromedullary bulbar region (arrow) indicating secondary olivary degeneration. **(C,D)** Axial diffusion weighted imaging and corresponding apparent diffusion coefficient map show normal diffusion within lesion (arrow) excluding acute ischemia or abscess recurrence. **(E)** Axial T_2_WI at the cerebellopontine level depicts patchy hypointensity in the right dentate nucleus (arrow). **(F)** Axial susceptibility-weighted imaging (SWI) confirms hemosiderosis in the right dentate nucleus. **(G)** Three-dimensional reconstruction of diffusion tensor imaging, via *syngo*. MR Neuro 3D (Siemens, Erlangen, Germany), reveals marked fiber tract disruption in the left olivary nucleus consistent with transsynaptic degeneration. **(H)** FLAIR sequence reveals persistent T_2_ hyperintensity in enlarged left anteromedullary bulbar (arrow). **(I)** SWI demonstrates persistent hypointensity in the right dentate nucleus (arrow) suggestive of hemosiderin deposition.

**Figure 3:**
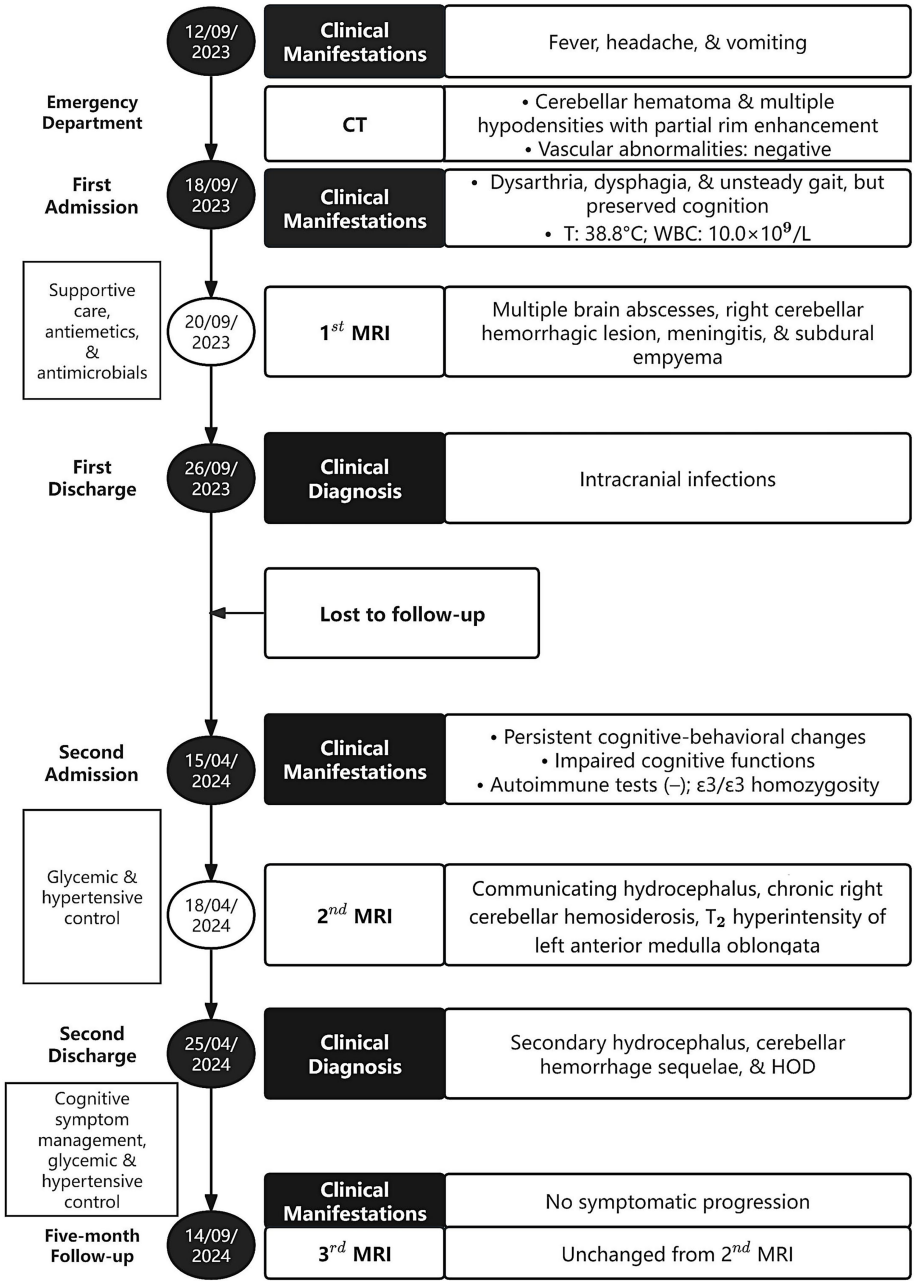
Timeline of clinical events of the patient. CT, computed tomography; T, temperature; WBC, white blood cells; MRI, magnetic resonance imaging; HOD, hypertrophic olivary degeneration.

Seven months post-discharge, the patient presented with persistent cognitive-behavioral changes (memory deterioration and personality alteration), marked by disorganized speech, tangentiality, and emotional lability with irritability. Activities of Daily Living preserved without functional impairment, urinary incontinence, dysphagia, or dysarthria. External hospital MRI indicated hydrocephalus, leading to readmission. Neurological exam confirmed isolated cognitive decline without motor, sensory, or cranial nerve deficits. Cognitive assessments showed the Mini-Mental State Examination score of 27/30, Montreal Cognitive Assessment score of 16/30, and Clinical Dementia Rating score of 0.5/3. Lab tests ruled out autoimmune encephalitis, showing normal immunoglobulins (Ig) (IgA, IgG, and IgM) and complement profiles (C3 and C4), and negative antibodies (anti-double-stranded DNA, antinuclear, and extractable nuclear antigen). Apolipoprotein E genotyping demonstrated ε3/ε3 homozygosity. Multimodal 3 T MRI revealed communicating hydrocephalus, hemosiderosis of the right cerebellar hemisphere, and a patchy hyperintensity on T_2_-weighted imaging of the left anterior medulla oblongata (Figures 2A–G). Lumbar drainage and gait assessment excluded normal pressure hydrocephalus. Differential diagnoses also excluded medullary infarction (absence of diffusion restriction on DWI) and amyotrophic lateral sclerosis (atypical topography). The final diagnosis was secondary hydrocephalus with cerebellar hemorrhage sequelae and HOD. Management included symptomatic support, i.e., glycemic control (glibenclamide 30 mg qd and acarbose 50 mg q8h) and antihypertensives (nifedipine 30 mg qd and amlodipine 5 mg qd). Post-intervention symptom relief was achieved. The patient was discharged with cognitive symptom management and ongoing glycemic and hypertensive control. At 5-month follow-up, head 3 T MRI confirmed persistent lesions (Figures 2H,I) without symptom progression or cognitive deterioration. The timeline of clinical events is summarized in Figure 3.

## Discussion

HOD is a specific transsynaptic neurodegenerative disorder characterized by secondary hypertrophy of the inferior olivary nucleus (ION) following GMT injury. It occurs across all age groups (Ogut et al., 2023), with a potential male predominance (Marrakchi et al., 2024). The GMT is a neural circuit composing the cerebellar dentate nucleus, contralateral red nucleus, and contralateral ION. Afferent fibers originate in the dentate nucleus, traverse the superior cerebellar peduncle to reach the contralateral red nucleus, descend to the ION via the central tegmental tract, and ultimately connect back to the contralateral dentate nucleus through the inferior cerebellar peduncle (Van Eetvelde et al., 2016; Steidl et al., 2022). As a pivotal cerebellar-brainstem connection, the GMT integrates cerebellar circuitry into broad functional networks, facilitating fine motor control and autonomously regulated movements (Van Eetvelde et al., 2016). Lesions within this circuit disrupt multi-regional signal transmission, leading to movement, speech articulation, and swallowing dysfunctions (Choi, 2016; Fleet et al., 2020). The afferent ION pathways include excitatory fibers and inhibitory dentate-olivary fibers. Damage to the GMT (e.g., dentate or red nucleus lesions), results in loss of inhibitory GABAergic fibers and over-enhanced excitatory afferent impulses, causing persistent excitation of inferior olivary neurons (Akar et al., 2008; Shaikh et al., 2010; Gatlin et al., 2011; Gao et al., 2022). This triggers compensatory neuronal and glial hyperplasia, forming the hallmark pathological features of HOD: ION enlargement and MRI-detected signal abnormalities. Notably, the efferent ION fibers projecting to the contralateral dentate nucleus do not participate in HOD pathogenesis (Behzadi et al., 2021), while the red nucleus plays a relatively minor role in the GMT loop (Jang and Borruat, 2014).

The most common etiology of HOD is cerebrovascular disease, including hemorrhage, infarction, arteriovenous malformation, etc. It may also be secondary to traumatic brain injury, brain tumors, multiple sclerosis, intracranial surgery and other factors (Sabat et al., 2016; Gao et al., 2022). In particular, mitochondrial dysfunction may also lead to bilateral HOD (Gao et al., 2022). HOD secondary to infection is rare. In brain abscesses, an overactive immune response releases cytokines such as tumor necrosis factor-*α* and interleukin-6, which can directly damage the brain parenchyma. Concurrently, cytokines recruit and activate inflammatory cells and glia via a positive feedback loop, which will lead to a continuous inflammatory response that further expands the area of parenchymal injury. A study has demonstrated that interleukin-6 drives the progression of neurodegenerative diseases by modulating acid-sensing ion channel 1a. This mechanism induces sustained neuroinflammation, calcium overload and aberrant signal transduction, ultimately leading to impaired synaptic function and triggering neuronal degeneration and death (Castellanos et al., 2024). In addition, the inflammatory response will also contribute to the breakdown of the blood–brain barrier (Kielian, 2004). The resulting loss of selective permeability allows peripheral immune cells to infiltrate the brain parenchyma, thereby exacerbating neuroinflammation (Tastan and Heneka, 2024). Another study indicates that astrocytic gap junctions, which transport small molecules, exhibit impaired function in peri-abscess regions (Karpuk et al., 2011). Overall, neuroinflammation ultimately manifests as a broad spectrum of severe clinical consequences, including memory impairment and cognitive dysfunction, impact on neural oscillations, pain and sensory disturbances, mood disorders, and cellular senescence and aging (Tastan and Heneka, 2024). In this case, the CT scan before the first admission showed a hemorrhagic focus rather than the usual caseous necrotic tissue inside the brain abscess. Brain abscess co-occurring with hemorrhage is rare, and the precise pathophysiological relationship between the two is unclear. Proposed mechanisms include: (1) Intracerebral hemorrhage may create a favorable microenvironment for bacterial colonization, thereby promoting local abscess formation. (2) During abscess capsule formation or progressive expansion, the integrity of cerebral vessels can be disrupted, impairing perivascular thrombosis and potentially leading to vascular rupture (Eza et al., 2024). (3) Pathogen infection can damage the vascular walls, resulting in the formation of a mycotic aneurysm that is prone to rupture and subsequent hemorrhage (Mencinger et al., 2021). (4) The mass effect of brain abscess and associated perifocal edema can elevate intracranial pressure, which may contribute to secondary vascular compromise. Elevated intracranial pressure causes rupture of newly formed blood vessels post-inflammation, allowing blood to flow into the necrotic center of the abscess with lower pressure (Orita et al., 1987). Blood and its derivatives can stimulate the GMT loop, particularly when the dentate nucleus is involved, leading to overexcitation of the ION (Tartaglione et al., 2015).

The typical clinical manifestation of HOD is palatal myoclonus, which is anatomically linked to the central tegmental tract and the nucleus ambiguus of the vagus. The latter controls the muscle groups involved in jaw movement. It is noteworthy, however, that palatal myoclonus is not pathognomonic for HOD (Wang et al., 2019). HOD may lead to oculopalatal tremor, linked to enhanced electrical coupling between ION cells (Shaikh et al., 2010). It can also cause Holmes tremor, which is associated with damage to extrapyramidal pathways (Dogan, 2020). In addition, HOD can also manifest with other symptoms, including ataxia, dysarthria, and diplopia (Dogan, 2020; Gao et al., 2022). Although HOD is usually accompanied by significant clinical symptoms, some cases, like this one, may lack specific clinical manifestations. Retrospective analysis indicates that nearly half of HOD cases are idiopathic, often being identified incidentally through neuroimaging (Van Eetvelde et al., 2016). Radiographic features like ION enlargement and signal abnormalities offer key evidence for early HOD diagnosis, which is particularly valuable in asymptomatic or paucisymptomatic patients.

Precise lesion localization within the GMT predicts olivary degeneration laterality. Specifically, when the lesion is located in the dentate nucleus or the superior cerebellar peduncles, degeneration is seen on the contralateral side; when the lesion involves the red nucleus and the central tegmental tract, degeneration is seen on the same side; when the lesions affect the bilateral dentate nucleus, the bilateral red nuclei, the bilateral central tegmental tract, or both the superior cerebellar peduncle and the central tegmental tract, degeneration can be seen on both sides (Gatlin et al., 2011; Cachia et al., 2013; Gao et al., 2022). Furthermore, bilateral HOD can also be triggered by damage to the left or dorsal midline of the pons of the GMT (Zheng et al., 2022). In this case, the lesion in the right cerebellar dentate nucleus blocked its inhibitory regulation of the left ION, resulting in subsequent neuronal overexcitation and hypertrophy of the left ION. This pathological change is closely related to the lesion site of the GMT system and the complex anatomical relationship between the GMT system and the ION.

The affected ION undergoes a series of pathological changes throughout the disease course, which can be categorized into six stages: (1) Within 24 h, no significant changes are observed; (2) Within 2**–**7 days or longer, degeneration of the ION manifests; (3) After 3 weeks, neuronal hypertrophy develops with mild ION enlargement; (4) Approximately 8.5 months later, both neurons and astrocytes undergo hypertrophy; (5) Around 9.5 months, pseudo-hypertrophy of the ION becomes apparent; and (6) After several years, neuronal loss occurs with concurrent ION atrophy and degeneration (Goto and Kaneko, 1981). Gliosis-induced increased water content and structural abnormalities manifest as T_2_-hyperintense signals within the ION on MRI (Kitajima et al., 1994). Correspondingly, MRI characteristics evolve with disease course: (1) During the initial 6 months, T_2_-weighted and proton density-weighted imaging demonstrate signal hyperintensity without concomitant olivary hypertrophy; (2) Subsequently, both signal abnormality and volumetric enlargement become evident; (3) After 3**–**4 years, hypertrophy gradually regresses while T_2_ hyperintensity often persists for years (Goyal et al., 2000). In this case, MRI obtained after the second admission (about 7 months post-onset) and at subsequent follow-up revealed persistent hemosiderin deposition in the right cerebellar hemisphere, alongside T_2_ hyperintensity and hypertrophic changes in the left ION. These imaging features correspond precisely to the HOD characteristics of pathological stage 3**–**5 and radiographic stage 2, as defined by the temporal progression of ION hypertrophy and signal abnormalities. This concordance between clinical imaging and pathological staging provides robust diagnostic validation, particularly in cases where classic symptoms may be absent.

The diagnosis of HOD mainly relies on three pillars: classic clinical manifestations, identification of primary GMT-disrupting lesions, and unilateral or bilateral ION enlargement with T_2_ hyperintensity (Yang et al., 2020). Critically, this case exemplifies atypical presentations where patients may lack characteristic symptoms, underscoring the indispensable role of MRI in diagnosis. Among MRI modalities, T_2_-weighted imaging (T_2_WI) demonstrates superior specificity for manual analysis, while diffusion tensor imaging (DTI) exhibits enhanced sensitivity and DWI offers differential clues (Steidl et al., 2022). DTI parameters provide quantitative biomarkers: reduced fractional anisotropy indicates neuronal fiber loss and demyelination, while increased mean diffusivity suggests decreased cellular density (Alexander et al., 2007). This case utilized advanced three-dimensional post-processing techniques to map white matter fibers through voxel-wise analysis of fiber direction continuity (Lazar, 2010). DTI tractography application, now well-established for detecting white matter abnormalities (Gatto et al., 2024), enables direct visualization of ION structural damage, providing conclusive imaging evidence for HOD diagnosis.

To review the literature, we performed a decade-long analysis of HOD cases secondary to intracranial infection (Table 1), highlighting common characteristics. A total of five cases spanned ages 7**–**62 years, demonstrating susceptibility across all life stages, with causative agents including *Toxoplasma gondii* (Sabat et al., 2016; Behzadi et al., 2021), *Listeria monocytogenes* (Oh, 2018), *Streptococcus intermedius* (Tonnu et al., 2022), and *Orientia tsutsugamushi* (Datta et al., 2024). All patients received pathogen-specific antimicrobial therapy. Clinical manifestations of HOD centered on coordination dysfunction, notably palatal tremor and ataxia. It was detected typically 1.5**–**4 months post-infectious onset (via follow-up MRI for primary infection or prolonged cryptic symptoms), yet none initially considered HOD, contributing to diagnostic delays. MRI consistently showed T_2_-hyperintense lesions, with bilateral distribution in two cases (Behzadi et al., 2021; Datta et al., 2024) and unilateral lesions in three (Sabat et al., 2016; Oh, 2018; Tonnu et al., 2022). Outcomes varied, with most achieving clinical/radiological stabilization or partial improvement, though one case lacked outcome reports and another required hospice due to comorbidity. Notably, Tonnu et al. (2022) described HOD from bacterial abscesses with early symptoms, contrasting our incidental imaging without classic signs. This report documents hemorrhagic cerebellitis-associated HOD, a rare phenotype, while both studies confirm fiber tracking’s utility in axonal injury visualization across neurological disorders. Together, these cases emphasize the necessity for heightened HOD vigilance post-infection, with multimodal MRI enabling early intervention through combined antimicrobial and symptomatic management.

**Table 1:** Summary of clinical characteristics of HOD cases with infectious etiologies.

Authors, year	Age, sex	Pathogen	Follow-up duration^a^	Clinical manifestations upon HOD development	Imaging features	Non-Anti-infective interventions^b^	Outcomes
Sabat et al. (2016)	62 y, Male	*Toxoplasmosis*	3 m	Recurrent falls, weakness, and poor coordination	Right HOD	Not reported	Acute inpatient hospice for uncontrolled HIV
Oh (2018)	48 y, Female	*Listeria monocytogenes*	4 m	Lateral gaze palsy, left eyelid ptosis, dysarthria, palatal tremor, and vocal cord oscillation	Left HOD	Clonazepam and baclofen	No response
Behzadi et al. (2021)	54 y, Female	*Toxoplasmosis*	6 w	Not reported	Bilateral HOD	Not reported	Radiologically improved for infectious lesions
Tonnu et al. (2022)	58 y, Female	*Streptococcus intermedius*	4 m	Dysarthria, palatal myoclonus, spastic gait, coordination dysfunction, and Romberg sign	Right HOD	Rehabilitation	Symptomatically improved
Datta et al. (2024)	7 y, Male	*Orientia tsutsugamushi*	2 y	Ataxia and signs of cerebellar involvement	Bilateral HOD	Amantadine and symptomatic therapy	Clinically stable

HOD, hypertrophic olivary degeneration; y, years; m, months; w, weeks; HIV, human immunodeficiency virus. ^a^Duration from the initial onset of infection to the first detection of HOD. ^b^To address the interventions specific to HOD, anti-infective agents are not reported.

Currently, no curative treatment exists for HOD. Symptomatic treatment includes clonazepam, levodopa, or other dopaminergic agents, with deep brain stimulation as a potential option (Wang et al., 2019). Cerebellospinal transcranial direct current stimulation combined with treadmill training shows promise in improving ataxia (Wang et al., 2024). In this case, diagnosis was achieved solely through imaging, precluding targeted intervention. However, the absence of symptoms necessitates vigilant follow-up to detect future manifestations. Preoperative neuroimaging evaluation of GMT anatomical risk is crucial for preventing iatrogenic HOD during brainstem procedures (Yun et al., 2013). Intraoperative GMT preservation and postoperative monitoring are essential.

In conclusion, this case expands the HOD etiology to include hemorrhagic cerebellitis, emphasizing that clinicians must consider HOD in post-infectious contexts even in the absence of classic symptoms. Early multimodal MRI (fluid-attenuated inversion recovery and DTI) enables timely diagnosis, while proactive neurocognitive surveillance may improve long-term outcomes. Further research into pathogen-specific mechanisms and neuroprotective strategies is imperative to mitigate this debilitating complication.

## Data Availability

The original contributions presented in the study are included in the article/Supplementary material, further inquiries can be directed to the corresponding authors.
