# Supplementary material for: Hypertrophic olivary degeneration secondary to brain abscess: a case report and literature review
Source: Front Hum Neurosci. 2025 Oct 28;19:1674100. doi: 10.3389/fnhum.2025.1674100 (PMC12602417; doi:10.3389/fnhum.2025.1674100)
Supplement: Supplementary file 1 [file Data_Sheet_1.pdf]

CARE
case report guidelines

CARE Checklist of information to include when writing a case report

(cc) BY-NC-ND

Topic	Item	Checklist item description	Reported on Line
Title	1	The diagnosis or intervention of primary focus followed by the words "case report"	55-62
Key Words	2	2 to 5 key words that identify diagnoses or interventions in this case report, including "case report"	99-100
Abstract (no references)	3a	Introduction: What is unique about this case and what does it add to the scientific literature?	77-79/91-96
	3b	Main symptoms and/or important clinical findings	80-87
	3c	The main diagnoses, therapeutic interventions, and outcomes	82-85/87-90
	3d	Conclusion—What is the main "take-away" lesson(s) from this case?	91-96
Introduction	4	One or two paragraphs summarizing why this case is unique (**may include references**)	111-123
Patient Information	5a	De-identified patient specific information.	128
	5b	Primary concerns and symptoms of the patient.	128-130/153-156
	5c	Medical, family, and psycho-social history including relevant genetic information	130-131
	5d	Relevant past interventions with outcomes	130-131
Clinical Findings	6	Describe significant physical examination (PE) and important clinical findings.	135-141/153-156
Timeline	7	Historical and current information from this episode of care organized as a timeline	457-510
Diagnostic Assessment	8a	Diagnostic testing (such as PE, laboratory testing, imaging, surveys).	130-146/156-175
	8b	Diagnostic challenges (such as access to testing, financial, or cultural)	87-88
	8c	Diagnosis (including other diagnoses considered)	146/175-177
	8d	Prognosis (such as staging in oncology) where applicable	182-185
Therapeutic Intervention	9a	Types of therapeutic intervention (such as pharmacologic, surgical, preventive, self-care)	146-150/177-179
	9b	Administration of therapeutic intervention (such as dosage, strength, duration)	146-151/177-179
	9c	Changes in therapeutic intervention (with rationale)	-
Follow-up and Outcomes	10a	Clinician and patient-assessed outcomes (if available)	-
	10b	Important follow-up diagnostic and other test results	182-184
	10c	Intervention adherence and tolerability (How was this assessed?)	151-152
	10d	Adverse and unanticipated events	-
Discussion	11a	A scientific discussion of the strengths AND limitations associated with this case report	620-635/667-670
	11b	Discussion of the relevant medical literature **with references**.	636-661
	11c	The scientific rationale for any conclusions (including assessment of possible causes)	382-438/590-616
	11d	The primary "take-away" lessons of this case report (without references) in a one paragraph conclusion	674-681
Patient Perspective	12	The patient should share their perspective in one to two paragraphs on the treatment(s) they received	-
Informed Consent	13	Did the patient give informed consent? Please provide if requested	Yes ☑ No ☐
