# Supplementary material for: Hypertrophic olivary degeneration secondary to brain abscess: a case report and literature review
Source: Front Hum Neurosci. 2025 Oct 28;19:1674100. doi: 10.3389/fnhum.2025.1674100 (PMC12602417; doi:10.3389/fnhum.2025.1674100)
Supplement: Supplementary file 2 [file Data_Sheet_2.docx]

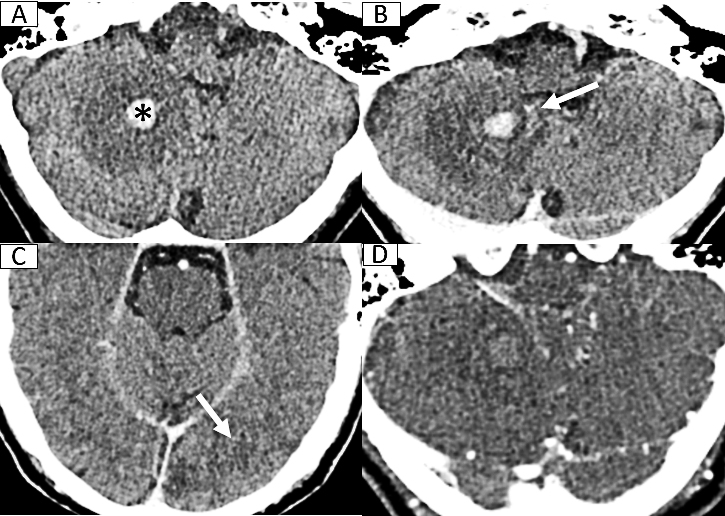


**Supplementary Figure S1. Head CT before first admission**

(A) Non-contrast axial CT (medulla oblongata level) reveals a hyperdense hematoma (asterisk) in the right cerebellar hemisphere with surrounding hypodense cerebral edema area. (B) Contrast-enhanced CT (the same level of A) shows mild peripheral enhancement (arrow) within the edema region. (C) Contrast-enhanced CT (pontine level) exhibits absence of enhancing hypodense lesion (arrow) in the left occipital lobe. (D) CT angiography (the same level of A) demonstrates no vascular abnormalities in the right cerebellar hemisphere.
